# A Qualitative Study of Patients’ Experiences, Enablers and Barriers of Rheumatic Heart Disease Care in Uganda

**DOI:** 10.5334/gh.1181

**Published:** 2023-02-23

**Authors:** Hadija Nalubwama, Jafesi Pulle, Jenifer Atala, Rachel Sarnacki, Miriam Nakitto, Rebecca Namara, Andrea Beaton, Rosemary Kansiime, Rachel Mwima, Emma Ndagire, Emmy Okello, David Watkins

**Affiliations:** 1 Uganda Heart Institute, Kampala, Uganda; 2 Children’s National Hospital, Washington DC, USA; 3 Department of Global Health, University of Washington, Seattle WA, USA; 4 Cincinnati Children’s Hospital Medical Center, Cincinnati OH, USA; 5 Department of Pediatrics, University of Cincinnati College of Medicine, Cincinnati, OH, USA; 6 Division of General Internal Medicine, University of Washington, Seattle, WA, USA

**Keywords:** rheumatic fever, rheumatic heart disease, Uganda, public health systems research

## Abstract

**Introduction:**

Rheumatic heart disease (RHD) remains a significant public health problem in countries with limited health resources. People living with RHD face numerous social challenges and have difficulty navigating ill-equipped health systems. This study sought to understand the impact of RHD on PLWRHD and their households and families in Uganda.

**Methods:**

In this qualitative study, we conducted in-depth interviews with 36 people living with RHD sampled purposively from Uganda’s national RHD research registry, stratifying the sample by geography and severity of disease. Our interview guides and data analysis used a combination of inductive and deductive methods, with the latter informed by the socio-ecological model. We ran thematic content analysis to identify codes that were then collapsed into themes. Coding was done independently by three analysts, who compared their results and iteratively updated the codebook.

**Results:**

The inductive portion of our analysis, which focused on the patient experience, revealed a significant impact of RHD on work and school. Participants often lived in fear of the future, faced limited childbirth choices, experienced domestic conflict, and suffered stigmatization and low self-esteem. The deductive portion of our analysis focused on barriers and enablers to care. Major barriers included the high out-of-pocket cost of medicines and travel to health facilities, as well as poor access to RHD diagnostics and medications. Major enablers included family and social support, financial support within the community, and good relationships with health workers, though this varied considerably by location.

**Conclusion:**

Despite several personal and community factors that support resilience, PLWRHD in Uganda experience a range of negative physical, emotional, and social consequences from their condition. Greater investment is needed in primary healthcare systems to support decentralized, patient-centered care for RHD. Implementing evidence-based interventions that prevent RHD at district level could greatly reduce the scale of human suffering. There is need to increase investment in primary prevention and tackling social determinants, to reduce the incidence of RHD in communities where the condition remains endemic.

## Introduction

Rheumatic heart disease (RHD) has largely disappeared from high-income countries but remains a significant public health problem in many low- and middle-income countries. The condition affects about 40 million people worldwide and causes about 300,000 deaths each year [1]. The persistence of severe and fatal RHD reflects the failure of primary healthcare systems to implement evidence-based measures like treatment of streptococcal pharyngitis and continuous antimicrobial chemoprophylaxis for individuals with a history of RHD or its antecedent, rheumatic fever (RF). Indigenous and other marginalized populations tend to be disproportionately affected [1,2,3,4,5,6,7,8]. Most individuals with RHD are on multiple chronic daily medications, and many require cardiac surgery at some point in their lives, so they must interact with the healthcare system frequently and at multiple levels, from primary to specialty care. Further, they have a shorter life expectancy as compared to the general population.

Among the countries where RHD remains endemic, few have formal health programs that integrate prevention and management of RHD. For example, in Uganda RHD is the most commonly diagnosed acquired heart disease among children and young adults [9], but prevention and treatment services are highly centralized due to lack of existing health system capacity at the community level [10]. Because of public underinvestment in RHD prevention and care, people living with RHD potentially face numerous challenges, from disabling symptoms that affect work and school to the high costs of medications, surgical procedures, and travel, which usually need to be paid for out of pocket [11,12].

There have been several recent efforts to tackle RHD in endemic countries like Uganda. In 2015, African Union governments adopted the recommendations of a communique developed by local clinical and public health experts [13], and in 2018 the World Health Assembly passed a resolution on RHD [14]. These political statements mandated ministries of health to improve timely, affordable, and reliable access to cost-effective interventions for acute RF and RHD. Recently, the Uganda Heart Institute (UHI) partnered with the Uganda Ministry of Health to develop a national RHD strategy that would support the implementation of the African Union and World Health Assembly resolutions. This strategy will expand access to care by (i) finding more people living with RHD (via screening) and (ii) providing RHD services in primary healthcare settings, close to where people live. While studies have been published on patient and community experiences with RHD preventive interventions [11,15], the Ministry of Health lacks local evidence on the impact of RHD itself and related healthcare in Uganda. The present study, which was part of this broader partnership, is a qualitative enquiry that sought to better understand the consequences of RHD among PLWRHD and their households, as well as identify the main barriers and enablers to care.

## Methods

This study adheres to the COnsolidated criteria for REporting Qualitative research (COREQ). Appendix 4 contains a completed version of the COREQ checklist for this manuscript [16].

### Study setting

Uganda is a low-income country in eastern sub-Saharan Africa with a population of about 44 million. More than 20% of Ugandans live below the national poverty line. In 2018, about one-fifth of the population lived below the national poverty line. Health expenditure in 2018 was US$ 43 per capita, 38% of which was paid for out-of-pocket [17]. The main provider of healthcare is the Ministry of Health, though many individuals seek care at private health facilities (and pay out-of-pocket), especially for services or medicines that are unavailable through the public sector [12]. The UHI, which is a publicly funded, semi-autonomous research and clinical center in the nation’s capital, Kampala, recently undertook a series of RHD epidemiological and health services research efforts in districts in northern and southwestern Uganda that were broadly representative of the economic, social, and demographic heterogeneity of the country. The UHI also maintains a National RHD Registry that captures the RHD-related medical records of patients who are diagnosed through echocardiography screening programs or following presentation to hospital with signs and symptoms of RHD. Over 3000 people living with RHD were enrolled as of March 2022.

In this study we conducted in-depth interviews among PLWRHD enrolled in the National RHD Registry who resided in the districts of Lira, Mbarara and Wakiso, three districts with considerable cardiovascular research infrastructure where substantial numbers of people living with RHD have been identified. Lira district is in the Northern Region and is located about 340 kilometers from Kampala. Mbarara district is in the Western Region and is located about 270 kilometers from Kampala. Both districts have significant rural populations. Wakiso district, located in the Central Region and adjacent to Kampala, is a largely urban district.

### Research team

The in-depth patient interviews were primarily conducted by one of the authors (HN), who is a public health officer with 12 years of experience (at the time of this study) conducting qualitative research. Three clinical research nurses (JA, RK, RM) with prior experience in qualitative research assisted with the HN- led interviews and conducted the subsequent interviews. All four interviewers were female, and at least one lived and worked in each of the districts where the interviews were conducted and was fluent in the local language.

### Study design and procedures

#### Overview and orientation

This study had two related objectives. The first was to understand the impact of RHD on people living with RHD, their households and families, and the broader community. For this objective, we took an exploratory approach that was influenced by grounded theory and used inductive coding techniques. The second objective was to identify barriers and enablers to RHD care from the perspective of people living with RHD. This objective had a more positivist orientation and used deductive coding techniques to map codes and themes to the socio-ecological framework.

#### Participant selection

Participants were recruited and interviews conducted between November 2018 and December 2019. Prior experience led us to develop a stratified purposive sampling strategy where our total sample was approximately equally distributed across the three districts mentioned above; within each district, we stratified our sample on the presence or absence of congestive heart failure (CHF), a marker of disease severity. We excluded children under age ten. We queried the National RHD Registry (n = 3114) for enrollees located at these three sites, with and without heart failure, and then used a random number generator to create a list of 64 eligible participants, oversampling in each stratum in case some eligible individuals declined to participate or were unable to be reached. From these, the first 36 participants who agreed to participate in the study were interviewed (using the rule of thumb in qualitative research that data from 30–40 participants would achieve saturation). In addition to the strata above, we sought to ensure that our final sample contained a balance of gender and age groups.

We used a standard recruitment script to contact eligible participants by telephone and invite them to participate. The script identified the caller as a member of the UHI research team, described the study’s objectives, and explained why the participant was eligible to be in the study. It then outlined the data collection procedures, described the compensation, and stressed that participation was voluntary and would not affect patient care. It was necessary for us to mention the latter because several of the subjects had a prior relationship with research team members through their participation in previous RHD research studies.

#### Data collection

We conducted interviews with the first 36 people living with RHD who agreed to participate in the study. No enrolled participants withdrew from the study. After informed consent was obtained, enrolled individuals were interviewed in the location of their preference (e.g., home or hospital premises). Only the interviewer, participant, and in some cases a second research nurse, were present during the interview.

Interviews were conducted in the participant’s preferred language and lasted 40 to 90 minutes. The lead interviewer asked all the questions, and in cases where the participant’s preferred language was different than the interviewer’s, an assisting research nurse translated questions and responses. The interviewer followed a semi-structured interview guide that contained a core set of questions and suggested prompts (see Appendix 1). All interviews were audio-recorded, and no identifying information was collected on the recordings. The recordings were then transcribed verbatim and translated into English. At the end of each interview, the recording was stopped, the participant thanked for their time, and a reimbursement of 20,000 Ugandan shillings (approx. US$ 5.6) was provided to cover time and travel costs.

Analysis of field notes (see Appendix 2) suggested thematic saturation had been achieved, so no additional subjects were recruited after the first 36 interviews were completed. We had originally planned to undertake member-checking to enhance rigor; however, these plans were cancelled in March 2020 due to the Covid-19 pandemic and a resulting suspension in most human-subject research in Uganda. We proceeded with the analysis and write-up of this study to provide timely evidence for the emerging national RHD strategy and time-sensitive research proposals.

#### Data analysis

Audio recordings were transcribed and translated into English for analysis by an independent experienced translator who was conversant in the local languages and in English. A second person cross-read the translations and confirmed that they were accurate interpretations of the local languages. The discrepancies during the data translation were resolved by consensus. Three team members (HN, JP, and RN) participated in the coding and analysis. Before coding was attempted, the analysts read the transcripts several times to ensure familiarity with the data. Transcript management and coding were done in Atlas.ti, version 8.4.25 [18].

As mentioned previously, we did two types of analysis related to the two different study objectives. The first analysis used a grounded-theory approach to understand the experiences of people living with RHD. Open, axial, and sequential coding procedures were used to develop clusters, themes, and subthemes from an initial coding of five transcripts. Each transcript was coded independently by the three team members (HN, JP, and RN), two of whom did not participate in the original interviews. The coders compared the codes they had developed independently and refined their lists until consensus was achieved and a final code book (Appendix 3) developed. Any discrepancies in the coding were resolved by group consensus.

The second analysis used a deductive approach that mapped all codes and themes related to healthcare barriers and enablers to the socio-ecological model [19]. As part of this exercise, we looked at the variation in themes by location and severity of disease, hypothesizing that both factors would lead to substantial variation in the type and relative frequency of themes identified.

### Ethics statement

The study was approved by the institutional review boards of Makerere University School of Medicine in Uganda (REC REF 2018-082), and the University of Washington in the United States (STUDY00002855). We sought clearance from Uganda National Council for Science and Technology (SS 5081) and permission to conduct the study from the relevant health authorities in all districts.

## Results

We recruited 12 participants from each of the three districts. Participants’ median age was 24.5 years and ranged between 11 and 55 years. Two-thirds of the respondents were female. Only one-third had some form of employment, and half had attained only primary level of education. Table 1 provides demographic information for the participants.

**Table 1:** Demographic characteristics of participants.


VARIABLE	VALUE

Age (median, range)	24.5 (11–55)

Female sex	22 (64%)

Presence of CHF	17 (47%)

Educational attainment	

Primary	18 (50%)

Secondary	6 (17%)

Tertiary	12 (33%)

Occupation	

Unemployed	15 (42%)

Employed	13 (36%)

In school	8 (22%)

Marital status	

Not married	18 (50%)

Married	16 (44%)

Widowed	2 (6%)


### Experiences of people living with rheumatic heart disease

#### Domain 1: Impact on the RHD patient

*Theme 1a: Interference with work, school, and future plans*

Study participants, especially those with congestive heart failure, expressed frustration over their limited ability to engage in their preferred work. They attributed these actions primarily to physical weakness and frequent hospital visits.

‘Now this disease has disturbed me a lot, earlier I would go to my banana plantation and work, in two weeks I would be selling like 15 matooke [local name for banana], but now when I stopped working…, it’s not easy even to get what to eat. Sometimes I would ride my bicycle go to the market and repair shoes, in a week I would be making about 20,000ugshs or 30,000ugshs [about US$ 5.6 to 8.4]. But now I left all that, my income reduced to 8,000ugshs [about US$ 2.2].’ CHF 012 MBARARA

Younger participants reported missing or dropping out of school, which greatly interfered with their career plans. Many school days were missed because of needing to travel to (often distant) health facilities to receive their routine, monthly preventive antibiotics.

‘Sometimes while at school, I get called that I am needed in Mulago [national referral hospital in Kampala], and sometimes when it’s close to examination period I end up missing the exams and go[ing] to the hospital, [and] by the time I come back, I find when they have already finished the exams.’ CHF 009 MBARARA

‘This year am supposed to go and do my diploma, but the RHD has prevented me from going ahead.’ Non-CHF 005 LIRA

Almost all study respondents reported unfulfilled dreams due to RHD and compared themselves unfavorably with their peers.

‘I thought I would be somebody in future, but now it is hard… I thought I would do some work that would also help me and other people as well. Now my future plans have been shuttered…’ Non-CHF 003 LIRA

*Theme 1b: Living in constant fear of the future*

Participants revealed that living with RHD sparks off constant fear and worry, especially about the possibility of RHD treatment failure, which would result in death and failure to meet family obligations.

‘At times I think… there’s a limit for the mechanical valve… Can a muscle grow and affect the valve to stop working or not? I have fears that sometime this disease… might overpower the strength of the medicine.’ Non-CHF 006 Wakiso

Participants with families expressed their constant worry about dying and leaving their children helpless.

‘I am always lost in deep thought. When [I] look at [my] kids and the fact that I was [once] able to do something for myself but now am just [here]… I worry that I am going to die and leave my children.’ CHF 001 WAKISO

*Theme 1c: Limited birth choices and experiencing interrelationship conflicts*

Childbearing and family formation is a very important part of Ugandan culture. Adults are generally expected to marry and become parents, and those who cannot bear children often suffer from lower social standing in their families and community [20]. Because of this, female participants expressed despair regarding their inability to have the desired number of children due to their RHD, which carries high risk of maternal and neonatal mortality [21].

‘I might not have a normal delivery, and then I am scared that my baby might not be all that normal. Because of [RHD]. When I went to [a] doctor, he also told me the same story. He [said], “you are most likely to produce a small baby.”’ Non-CHF 001 LIRA (pregnant female)

Participants also reported marital conflicts due to an inability to fulfill their (gendered) roles in the relationship. For example, one married woman with children expressed her worry about losing her marriage because she was unable to do housework due to her RHD. Similarly, a married man with RHD expressed his worry about being able to provide for his family and his wife leaving him for another man.

*Theme 1d: Stigmatization and loss of self-esteem*

Participants reported that RHD greatly affected their self-image and social standing. They often mentioned experiencing feelings of self-isolation because they tended to avoid public gatherings. Some participants were treated by their communities as though they had a contagious disease and were destined to die soon.

‘…people in the community don’t treat us well because they think that when you have a heart disease, maybe you looked for it and maybe you will transfer it to them.’ CHF 005 LIRA

#### Domain 2: Impact on families

*Theme 2a: Constant worry about the patient*

As suggested by the findings above, the emotional and psychological impact of RHD usually extended to participants’ family members.

‘They were worried that maybe I will not live longer… actually it’s my dad who is now [most] affected, because all the time he is stressed out. He even doesn’t want to hear that am not feeling well, because once he hears, he thinks maybe I am going to die.’ Non-CHF 001 LIRA

*Theme 2b: Financial crises*

RHD also significantly affected the financial standing of patients’ families. Importantly, some families were unable to pay for their children’s education due to medical expenses. In some families, older children dropped out of school to get jobs and help cover household expenses.

‘My brother is the one who was at school… [but] he stopped going to school to come and help my mum… [by taking on] some small [jobs] to get money that would be used to take me to the hospital.’ Non-CHF 007 LIRA

In some worst-case scenarios, families were unable even to cover essential expenses because of RHD treatment costs.

‘The money which my father and my mum get [was spent] to buy a drug. Some other time, we [go] to sleep without eating because there is no money [left].’ Non-CHF 006 LIRA

### Enablers of RHD care

#### Individual-level enablers

*Theme 3a: Positive attitude*

Participants reported that a positive attitude towards life and the desire to care for their families could, at times, support them in seeking care and adhering to RHD treatments.

#### Interpersonal- and community-level enablers

Social support, including support from partners, families, and communities, also enabled participation in RHD care.

*Theme 3b: Supportive spouses*

Supportive spouses were noted to be particularly helpful in providing an enabling environment for engaging in RHD care. Encouragement and reminders were cited as particularly effective at ensuring adherence and retention in care.

‘When my wife looks at my documents, she tells me that you have to return on this date and she cares a lot. My wife knows that I have a disease and she understands me well…’ Non-CHF 005 WAKISO

‘[My husband] encourages me. He says that ‘you will live as long as you are getting treatment’… he cares about me.’ CHF 001 WAKISO

*Theme 3c: Social support from peers*

Support from peers and community members was also frequently mentioned as an important enabler.

‘[Our friends] have been coordinating with my wife and giving her all the support we need. In fact, I have friends that bought some food and a few other things and then sent them to us.’ Non-CHF 005 WAKISO

Of course, the experience of strong community support was not universal, since other participants reported stigma (see theme 1d above).

*Theme 3d: Financial support*

Some participants also reported that their partners and communities came together and provided financial support, helping them to receive timely care (Non-CHF 013 MBARARA).

#### Structural (health system) enablers

*Theme 3e: Good patient care practices by healthcare providers*

Participants believed that they were more likely to access care and remain adherent to treatments when they were treated well by their providers.

‘I don’t want to miss my appointment, because [I know that] when I come on my appointment, I get good care.’ Non-CHF 004 LIRA

Providers who communicated well and provide information and counselling to patients were seen as particularly effective at enabling care.

### Barriers to RHD care

#### Individual-level barriers

*Theme 4a: Painful monthly Injections*

Monthly intramuscular injections of benzathine penicillin are necessary to prevent worsening of RHD. Participants reported these injections to be very painful and sometimes hindering treatment adherence. Some participants opted to wait until the injection site had healed before receiving another dose.

‘…the injection was so painful, I would take it one month [but not the next month] because it was so disturbing.’ CHF 012 MBARARA

*Theme 4b: Long distances and high costs of travel*

Almost all participants cited the cost of travel to receive their monthly injections as a major barrier to care. Because RHD services are centralized, most participants had to travel long distances for routine care, incurring substantial out-of-pocket costs on a regular basis.

‘When there is no money for transport, sometimes coming becomes hard, because when you [walk on] foot, you get so tired.’ Non-CHF 003 LIRA

*Theme 4c: Other competing needs*

RHD patients frequently faced a dilemma: receive RHD care and miss a day of work or school or go to work or school and forego RHD care. Respondents who feared serious consequences like job loss or jeopardized education often opted to forego care. This choice was especially common among individuals with milder disease.

‘Sometimes I have work to do [but have to] travel so far away; like I go to Kampala and I miss my appointment date…’ Non-CHF 013 MBARARA

*Theme 4d: The lack of encouragement to keep on medication*

A commonly reported reason for stopping treatment against medical advice was because participants felt better. In most cases, improvements in symptoms were short-lived, and without medication participants became sick again. This was especially the case among individuals with more severe disease.

‘In 2014, in the beginning till the end of the year, I didn’t take any medicines and I did not get injected, but I was well…but then I started feeling [sick again] in 2015.’ CHF 005 LIRA

One participant suggested lack of cultural sensitization about the importance of adhering to chronic medication use.

‘We don’t honor prescribed medication treatment, when you feel you are better, you just give up the medicine, it’s really in us, and I think we need sensitization about it.’ Non-CHF 015 MBARARA

*Theme 4e: Depression*

Though not commonly observed, some respondents reported symptoms of depression, including feelings of loss of hope and sadness that resulted in treatment default.

‘I refused to go and get injections, and I refused to swallow tablets because sometimes I can feel like am sad.’ CHF 014 MBARARA

*Theme 4f: Desperation*

Some of the participants saw no significant change in their condition despite taking medications and began to feel desperate. They suggested that having counselling sessions with health workers would be helpful in restoring their resolve to remain on medication. Similar suggestions came from respondents from all three districts, regardless of their disease severity.

‘[We RHD patients] lose hope, because if you are taking medication and you don’t see any change, it feels like nothing is being done… personally when I was taking that medication at first, it was just adding pain to me, so I had also decided not to continue with the medication, but [the health workers] counselled me, [and the other RHD patients] also need counselling.’ CHF 011 MBARARA

#### Interpersonal- and community-level barriers

*Theme 4g: Poor family and social support*

Whereas some participants were pleased with the support provided by spouses, families, and communities (see themes 3b-3d), other participants reported poor moral and social support. This left the them struggling physically, financially, and emotionally, making it difficult to navigate their care alone.

‘There are also nieces that we found lamenting, “when we want to treat him (RHD patient) for this, he falls sick with that! We even stopped supporting him with money”.’ Non-CHF 005 WAKISO

*Theme 4h: Stigma*

A few respondents, especially those from Wakiso, reported stigma among their peers and communities despite their resolve to manage their disease. These individuals felt as though they had little value and were thought to be destined to die soon, leading to isolation from their communities (see theme 1d).

‘…when you fall sick or get a problem, people start staying away from you. They start seeing you [as a] useless person; they were friends that had turned into family. “You are going to die,” [they say to] your face….’ CHF 001 WAKISO

*Theme 4i: Pressure to conform to societal norms*

Young women with congestive heart failure reported being pressured to bear multiple children, as is the norm in their communities [20]. This is despite this being contrary to the advice received from health workers underscoring the danger that pregnancy would impose on their lives, given the reduced functioning of the heart due to disease. Because pregnancy leads to hemodynamic changes, the already weak heart of the mother living with RHD would work harder to support the mother and fetus, resulting in higher risk of poor outcomes [22].

‘…the issue about childbearing disturbs [my husband] too. Whenever we visit his family, they keep on asking me when I will give birth again. So they are actually forcing me to give birth again, [and] it is hurtful.’ CHF 006 WAKISO

#### Structural (health system) barriers

*Theme 4j: High cost of RHD treatment*

The high cost of RHD care was a near-universal refrain. Although the Ministry of Health policies state that primary healthcare should be available free of charge, RHD is not prioritized in the national health strategy as other infectious diseases are, so there are insufficient resources to ensure truly free care. For example, individuals needing benzathine penicillin injections or congestive heart failure drugs are entitled to receive them for free in public healthcare facilities, but because of weak supply chains and stockouts (see theme 4k), these individuals often must go to private pharmacies, where they are expected to pay for medicines out-of-pocket [12].

‘If there is no money [to buy RHD drugs] then I have to wait until [I] get money. Sometimes… up to a month without medicine.’ Non-CHF 003 LIRA

Many respondents only took drugs they got either freely at the RHD clinic or those they could afford.

‘Yes, I miss [taking drugs] because of the problem of money for buying those drugs.’ Non-CHF 006 LIRA

The situation is even worse for the subset of individuals who require heart surgery since surgery is currently not guaranteed to be free. Respondents, especially those with severe RHD, were usually willing to undergo surgery when recommended, but they felt constrained by the very high out-of-pocket cost, which left them in a condition of despair.

‘I think to people like me who need (heart) surgery, I am a stay home mother but the price for surgery is high!’ CHF 001 WAKISO

While individuals tried going to their communities to raise funds for surgery, this was also extremely challenging, discouraging patients from even considering surgery as an option. These financial challenges caused further distress among households and peer networks.

‘They only told me about having an operation… when [my husband] heard about the operation, he was so disturbed. In fact he just told me [in an agitated tone], “it is up to you; stick to your medication until you die. I don’t have money for that operation”.’ CHF 004 WAKISO

In addition, the diets which were recommended for RHD patients (e.g., on anticoagulants) were reported to be unsustainable for those with meagre/no income.

‘We (RHD patients) have been informed a lot about what we are supposed to eat or what we are supposed to avoid, but still even after learning the diet that you are supposed to eat, you may sometimes lack the money to buy those foods.’ Non-CHF 001 WAKISO

*Theme 4k: The unavailability of medicines and healthcare providers at the public health facilities*

Participants in Mbarara and Lira districts (the two poorer districts included in this study) expressed their frustration and disappointment at having to travel long distances to health facilities, only to be find that medicines, diagnostics and health workers were unavailable. This frustration led some participants to abandon care since they were referred to private facilities where services were unaffordable.

‘If you come when you are feeling pain, sometimes your doctors are not around and you have to sit and wait, so you get fed up easily.’ CHF 011 MBARARA

‘…sometimes when you come to the hospital… you don’t find medicine and there is no money, [and] your troubles increase.’ Non-CHF 003 LIRA

*Theme 4l: Delays at the facility*

Delays at health facilities also hindered access to care. Many participants would miss a full day of work to attend their clinics, incurring other expenses like meals. This frustration was particularly pronounced in Lira, the district in our study that had the fewest health resources.

‘Patients are always many [waiting in the clinic], even where you thought you would spend two hours, you find you have spent the whole day, so before you leave home, you have to be prepared with transport, lunch money and other things.’ Non- CHF 013 MBARARA

‘Sometimes when you go and find so many people… the person working with the doctor comes and tells you that the doctor is tired and that people should go and come back the following day.’ Non-CHF 003 LIRA

*Theme 4m: Inadequate information and counselling given about RHD treatment*

Participants felt that information and counselling about RHD was inadequate. Participants reporting receiving wrong doses or missing their treatment for some time—in some cases, for a whole year.

‘In 2015, that’s when they told me that I have a rheumatic heart disease and it has no treatment, only to keep hygiene.’ Non-CHF 015 MBARARA

‘I didn’t know that I had to get [benzathine penicillin injection] every time I come here and now it feels like I have just started using it again.’ CHF 006 WAKISO (without medication for 1 year)

*Theme 4n: Failure to access RHD services in nearby health facilities*

Participants from all three districts expressed their frustration with the lack of RHD care in health facilities near them. One participant had a unique insight about the reasons for poorly-equipped facilities:

‘In my village [RHD] medicines are not available; they aren’t even stocked… my uncle [who sells medicines] said he can’t stock the medicines I take because they are very expensive yet very few people use them, [and when] they are stocked, they expire and he makes a loss.’ CHF 012 MBARARA

#### Role of RHD research clinics in enabling care: divergent themes

We must stress that our findings were influenced by concurrent research activities. The UHI had previously established RHD clinics at the local hospitals in Lira and Mbarara to provide care for people living with RHD who had been diagnosed in research contexts (e.g., through community-based prevalence studies). These clinics are linked to the National RHD Registry and are run by research nurses (including authors of this study) who are specifically trained to provide patient-centered RHD care. As a result of this arrangement, participants reported different healthcare experiences, depending on whether they were receiving treatment at these research clinics or in the community (i.e., general healthcare system). We identified some ‘divergent themes’ that emerged when individuals reflected specifically on their encounters at the RHD clinics.

*Divergent theme 1: Consistent availability of drugs at RHD clinics*

The RHD clinics at the Lira and Mbarara hospitals consistently provided most of the drugs for RHD patients for free.

‘I have never missed because every time I run out of drugs, I would find most of the drugs here the following month.’ Non-CHF 005 WAKISO

*Divergent theme 2: Availability of free tests*

Participants also mentioned that cardiac tests, especially echocardiography and electrocardiography, were available and free of charge at the RHD clinics, and that this was as an important enabler to their care.

‘…checkup is free because those tests are really expensive, like Echo… some other place it’s around 200,000ugshs [about US$ 56], but here they don’t ask for money… the INR [blood test] is 30,000ugshs [about US$ 8.4], but here it’s free.’ CHF 007 MBARARA

*Divergent theme 3: Availability of trained personnel*

The presence of health workers specifically trained in RHD care was also frequently mentioned as an enabler.

‘… the nurse is also responsible…. even when it rains you will find him in office to serve people.’ Non-CHF 005 WAKISO

### Variation of themes across districts and participant groups

Table 2 summarizes the major barriers and enablers to care that were common across all three districts and relevant to participants regardless of disease severity. The most frequently reported barriers to RHD care were long journeys and transport costs, high cost of treatment, and delays at the facilities. Peer support, consistent availability of drugs at RHD clinics, good care practices and counselling by health workers, and treatment reminders were the most frequently reported enablers of RHD care.

**Table 2:** Summary of key enablers and barriers to RHD care at various levels of the socioecological model.


LEVEL	ENABLERS	BARRIERS

**Individual**	Positive attitude	Painful monthly injections Long distances and high costs of travel Other competing needs Lack of encouragement to keep on medication Depression Desperation

**Interpersonal and community**	Supportive spouses Social support from peers Financial support	Poor family and social support Stigma Pressure to conform to societal norms

**Structural (health system)**	Stocking of medicines at nearby health centers Good care practices by healthcare providers	High cost of RHD treatment Unavailability of medicines and healthcare providers at public health facilities Delays at the facilities Inadequate information Failure to access RHD services at nearby facilities


**Figure 1:**
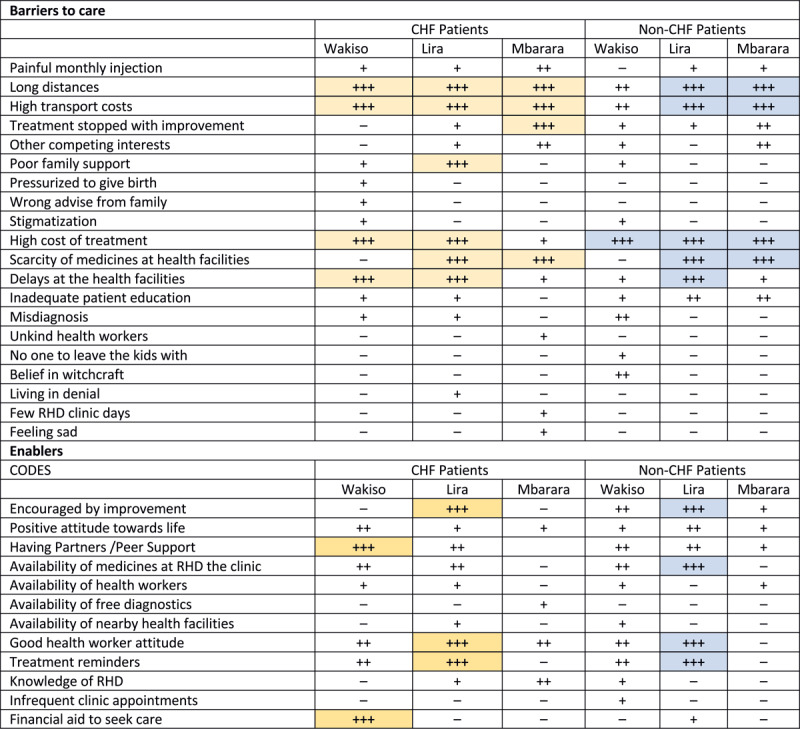
**Data display matrix showing the relative frequency of themes by setting and district.** Symbols indicate: – not noted in any transcripts; + noted in very few transcripts; ++ noted in significant number of transcripts; +++ noted in nearly all transcripts.

Figure 1 shows the relative frequency of these themes across districts and by severity of disease. Long travel distances to health facilities, high cost of travel, and high cost of treatment were common themes across geographical locations and disease severity groups. On the other hand, unavailability of drugs at facilities, unpleasant treatment by healthcare providers, and competing interests were more pronounced in Mbarara. In Wakiso, a more urban setting, pressure to give birth and stigmatization was more frequently mentioned. Non-CHF patients reported the lack of enough information and education on their disease state and misdiagnosis of their condition more frequently than CHF patients, who, in contrast, mentioned more frequently unpleasant treatment by healthcare providers.

Patients from Lira commonly reported divergent themes related to RHD research clinics, which included encouragement due to improvement as an enabler to treatment alongside good care practices by healthcare workers and treatment reminders. In the same way, social support and financial aid was more frequently reported by patients in Wakiso.

## Discussion

In this study, we interviewed 36 PLWRHD living in three regions in Uganda to gain an in-depth understanding of their experiences with the condition and the enablers and barriers to receiving healthcare. We found that patients and families experienced significant economic hardship related to out-of-pocket healthcare costs and limited work and school opportunities. We document frequent stigmatization and, among women of reproductive age, challenges around childbirth choices. Social support from spouses and peers, financial support, and positive encounters with health workers were noted to be the major enablers to care. Long travel distances to healthcare, high travel costs, delays in care, and pain related to treatment were noted to be the major barriers to care. We identified a few divergent themes among participants receiving care at research-established RHD clinics, which were relatively better resourced and staffed with nurses who had expertise in RHD management.

Few studies have been published on patient and family experiences living with RHD in limited resource settings. Our findings had some similarities to quantitative and mixed-methods studies from Rwanda [23] and Sudan [24]. The study from Rwanda found that long distances and long waiting times were major barriers to care. The study from Sudan found that reduced healthcare facility wait time, perception of adequate facility staffing, and high patient knowledge about RHD were significantly associated with increased adherence. The same study also found the inverse to be true, consistent with our study’s findings.

Our study corroborates findings from two prior studies in Uganda. A longitudinal study on adherence to benzathine penicillin found that residing near a health facility was associated with high adherence levels [25]. A qualitative study among persons with a history of RF, a risk condition for RHD, found that personal motivation, supportive family and friends, and a positive relationship with health care providers were key facilitators to medication adherence [11]. Respondents in our study were encouraged by respectful treatment by health workers and tended to avoid facilities with rude or non-welcoming health workers. Another study on community perspectives on prevention of RHD in Uganda also found that substantial costs associated with transportation and medication significantly hindered access to care [15].

Our study adds value to the previous literature from Rwanda, Sudan, and Uganda in two important ways. First, we focus on people living with RHD, many of whom are receiving cardiac medications or have undergone open heart surgery. These individuals inevitably have a different healthcare experience than, for example, persons with a history of RF, who tend to be healthier and are taking fewer medications. Second, we went outside the tertiary hospital setting to learn from the experiences of people living with RHD from diverse parts of the country, including very rural and very poor communities. We also note that the qualitative approach used in this study allowed us to gain a deep understanding of the lived experience with RHD, adding significant value to the literature and elevating the voices of patients and their families in research on health policy.

The findings of our research have several immediate implications for the Ministry of Health and the design of RHD programs and interventions in Uganda and other countries. First and foremost, the interviews underscore an urgent need to decentralize RHD services (especially diagnostics and routine antibiotic injections) from referral hospitals to facilities close to where people live. We recommend a ‘diagnonal’ approach to decentralization, integrating RHD services within the lower-level health facilities and using RHD as an opportunity to strengthen primary healthcare more generally [26]. This action would mitigate several barriers related to medical and non-medical costs, including time and money spent traveling to receive healthcare. In parallel with decentralization, the Ministry would need to strengthen supply chains for RHD-related medications so that patients do not have to pay for essential drugs at private pharmacies. These interventions concur with a recent study that found that a relatively small investment in health systems across African Union countries to integrate RHD care into primary care at lower-level facilities closer to patients would accrue early benefits, averting a considerable number of deaths [27]. The interviews also suggest that patient-led support groups, coupled with patient-facing provider communication and patient education interventions, could provide significant psychosocial support for treatment adherence. These could be modeled after successful RHD support groups for children [28] and ‘adherence clubs’ for persons with HIV who are on antiretroviral therapy [29]. Finally, we heard repeatedly from participants about the stigmas they face living with RHD. Standardized education and communication activities targeting health workers, family members, and the general community could be developed to improve awareness and understanding of RHD, allowing health systems and communities to provide better support to those living with the condition.

Despites its strengths, our study has some important limitations. Most notably, it was conducted in districts where concurrent research sites activities were being undertaken, leading to some divergent themes and challenges in interpreting the data. Additionally, we did not recruit any participants from the Eastern region of the country. This region is expected to have a high prevalence of RHD (due to high poverty rates, like in the Northern region), and has very limited healthcare resources. However, at the time of this study there were no participants in the Uganda National RHD Registry who were from this region, so our sampling frame was limited to participants from the other three regions.

## Conclusions

Despite several personal and community factors that support resilience, people living with RHD in Uganda experience a range of physical, emotional, and social consequences from their condition. Greater investment is needed in primary healthcare systems to support decentralized, patient-centered care for RHD. Implementing evidence-based interventions that prevent RHD at district level could greatly reduce the scale of human suffering. Additionally, there is need to increase investment in primary prevention and tackling social determinants of health, to reduce the incidence of RHD in communities where the condition remains endemic.

## Data accessibility Statement

Redacted versions of the in-depth interviews transcripts are available upon request.

## Additional File

The additional file for this article can be found as follows:

10.5334/gh.1181.s1 Appendices. Appendix 1 to 4.
