# Supplementary material for: A Qualitative Study of Patients’ Experiences, Enablers and Barriers of Rheumatic Heart Disease Care in Uganda
Source: Glob Heart. 2023 Feb 23;18(1):6. doi: 10.5334/gh.1181 (PMC9951636; doi:10.5334/gh.1181)
Supplement: Appendices. — Appendix 1 to 4. [file gh-18-1-1181-s1.pdf]

Appendix 1. Discussion guide for patient interviews

Objective: identify local barriers and enablers to RHD care

Selection criteria: patients on UHI RHD Registry, recruited in order to reflect variations in health behaviors according to

- Region (North, Central, West, East)
- Severity of disease (milder or more severe [i.e., waiting on surgery])
- Adherence to chronic medications (better vs. worse)

Turn on recorder.

Provide a copy of the informed consent form to the participant. Read it aloud word-for-word in case they cannot read. Provide an opportunity to ask questions. If they cannot provide written consent for any reason, tape-record their verbal consent.

Explain the ground rules:

- This is a study to determine how to provide better care for RHD
- There are no right or wrong answers
- Please be frank and share your opinion; the data we gather are confidential
- We will not link your identity to any of your comments in any reports we produce

Q1. Briefly, tell me about yourself

- Age, occupation (or level in school)
- Who lives at home with you (size of household, occupations of others)?
- Anybody besides you in your household use the health centres/hospital regularly for medical care?

Q2. Tell me about your RHD history

- In what year were you first diagnosed?

Formatted: Position: Horizontal: Center, Relative to: Margin, Vertical: 0", Relative to: Paragraph, Wrap Around

- When did you first start noticing symptoms? What were they?
- What made you seek care?
- How did you end up getting diagnosed with RHD (i.e., timeline, health providers and facilities visited from onset of symptoms to diagnosis)?

Q3. What's your understanding of this disease…

- What have you been told is the cause of RHD? By doctors? By others in the community?
- Ever heard of a link between sore throat and RHD? If yes, when and from whom?
- Ever been told about any treatments that can prevent or cure RHD or at least make your symptoms better?

Q4. Experience with healthcare system

- Initial treatments (i.e., when you were first diagnosed)?
- Improvement or worsening in symptoms since then?
- Current treatments?
- Still see healthcare providers regularly (i.e., at least twice a year)?
- Overall satisfaction with RHD care (**remind patient that this is confidential)
- Trust current providers to look after your needs?
- Any specific challenges to getting the care you think you need?
- In your opinion, what are the providers doing well (if anything)?

Q5. Impact on daily life

- Ease or difficulty getting to/from clinic or hospital
- Symptoms interfere with work/school/leisure? Quantify number of days of school or work missed over past month? Past year?
- Impact of disease on household finances

Q6. Current health-seeking behaviors

- What do you do when you start feeling sicker?
- Do you use any alternative sources of healthcare?

Formatted: Position: Horizontal: Center, Relative to: Margin, Vertical: 0", Relative to: Paragraph, Wrap Around

- What prevents you/would prevent you from keeping your appointments (if you miss them)? (**remind patient that it is common for people to miss appointments/medication doses, and we are trying to determine how to better support patients)
- What prevents you/would prevent you from taking your medicines/injections (if you miss them)?

Q7. Prospects for the future

- Hopes and dreams for school or work?
- How do these compare to your peers without RHD?
- Any worries about future health? Marriage? Work opportunities?
- How long do you think you'll have RHD? Expect it to get better, worse, or stay the same?

Thank participant for their time. Turn off recorder.

Provide opportunity for them to give feedback and ask questions.

Provide them with cash.

Fill out field notes once they have left.

Formatted: Position: Horizontal: Center, Relative to: Margin, Vertical: 0", Relative to: Paragraph, Wrap Around

Appendix 2. Prompts used for taking field notes

Setting of ~~focus group discussion~~ interview (geographic location/address and type of building/place):

Appearance and demeanor of group participants:

Demographic/socioeconomic composition (age groups, gender, occupations represented):

Notable behaviors or nonverbal cues (i.e., not captured in recording/transcript), presence of especially talkative or quiet participants (quantify both, if possible):

Any changes or adaptations of discussion topics, probes, etc. for this particular discussion?

Reflections:

- Did the discussion feel easy or difficult? Unnecessarily brief or lengthy?

- How did I perform during the interview? Was I successful in facilitating a balanced discussion where all members had opportunities to share their views?

- Do I note any potential biases or feelings of mine that came through during the interview and affected the conversation?

Formatted: Position: Horizontal: Center, Relative to: Margin, Vertical: 0", Relative to: Paragraph, Wrap Around

Appendix 3. Codebook for Patient Interviews

CODE	CODE DEFINITION
**Impact on Patients and their Families**	
Lack of support from school	Any mention of lack of understanding and consideration of RHD condition at school for a schooling RHD patient
Unable to continue with school	Any mention of anyone dropping out of school because they were unable to afford school fees
Lack of child care support	Any mention of having no support at home for the children to facilitate treatment seeking
Lack of reminders for treatment	Any mention of RHD patients lacking treatment reminder mechanisms in place for instance calls/SMS
Financial constraints	Any mention of general lack of money to pay for RHD treatment regulated costs
Job/ work affected due to illness	Any mention of challenges related to the changes in the work patients do
Pill burden	Any mention of challenges related the pill burden/swallowing many drugs over a long period of time and the related side effects
Worry to family	Mention of worry the patient’s family undergoes due to RHD
Spouse demands	Any mention of challenges related to the spouse influence in health actions patients should take
Stigma	Any mention of fear of being stigmatized by friends/siblings etc.
Marital challenges	Any mention of challenges related in marriage due to having RHD

Formatted: Position: Horizontal: Center, Relative to: Margin, Vertical: 0", Relative to: Paragraph, Wrap Around

Become burden to family	Any mention of a challenge related to being seen or feeling like the RHD patient is a burden to the family/partner
Inability to do gainful work	Mention of challenges related to difficulties in engaging in gainful work
Inability to do daily house chores	Any mention of a challenge related to difficulties in doing daily house chores
Financial crisis	Any mention of poor financial situation, and poverty resulting from RHD illness related causes
Body weakness	Any mention of physical body weakness due to RHD disease process
Many hospital visits	Any mention of inconvenience due to going to hospital several times
Abandonment by spouse	Any mention of being worried about by deserted by the partner
Isolation by peers	Any mention of not being involved in community events due to being isolated
Inability to have a child, start a marriage and stay married	Any mention around worrying about not having a child, family or marrying
**Barriers to RHD Care**	
Painful injections	Any mention of fearing the pain associated with Benzathine injection
Long distances traveled	Any mention of traveling long distances to the facilities
High cost of travel	Any mention of finding it hard to either raise money to travel to the Facilities
High cost of surgery	Any mention of lack of money enough money to access RHD surgery
High cost of drugs/ diagnostics	Any mention of high costs for either drugs or diagnostics as a barrier to treatment seeking

Formatted: Position: Horizontal: Center, Relative to: Margin, Vertical: 0", Relative to: Paragraph, Wrap Around

Other competing interests	Any mention of struggles faced in seeking treatment due to having other responsibilities like attending school, having to work etc.
Lack of knowledge on disease	Any mention of lack of awareness about the need to take drugs so as to stay healthy
Reluctancy to keep on treatment when feeling better	Any mention of patients not taking their medication when symptoms improve
Belief in witchcraft	Any mention of failure to access modern medical services due to belief in witchcraft as the possible cause of RHD and therefore seeking traditional remedies
Denial of disease	Any mention of denying the fact that have RHD
Delays at the facility	Any mention of delays in receiving any services at the health facilities
Feeling sad	Any mention of negative emotions that hinder treatment seeking
Few clinic days	Any mention related to not having enough clinic days.
Unkind treatment by health workers	Any mention of unfavorable /negative treatment from health workers experienced by patients
Delayed diagnosis	Any mention of long time between feeling sickness and getting a confirmed diagnosis of RHD
Misdiagnosis	Any mention of having wrong diagnosis made
Unavailability of medicines at nearby facilities	Any mention of RHD drugs being unavailable in patients' nearby health facilities
Absence of medical workers at the facilities	Any mention of the inability to find a health worker at the health facility where they sought healthcare for RHD
Inadequate family support	Any mention of RHD patients lacking treatment support from family members

Formatted: Position: Horizontal: Center, Relative to: Margin, Vertical: 0", Relative to: Paragraph, Wrap Around

Bad advice from family members	Any mention of patients being ill advised by family member
**Enablers to care**	
Determination to live	Any mention of adhering to health worker recommendations, treatment due to a personal resolve to stay healthy and lead a long life
Availability of trained health workers	Any mention of being motivated to seek RHD care due assurance of finding trained health workers at the health facility
Availability of medicines	Any mention of being motivated to seek RHD care due to availability of medicines in healthcare facilities
Long clinic appointment interval	Any mention of appreciating the 3 months treatment given which gives patients enough time to prepare for the next visit
Patient education	Any mention of motivation to seek and stay in RHD care due the adequacy of knowledge patients receive about their health condition
Financial assistance from programs	Any mention of having transport playing a big role in accessing care and keeping appointment. For instance, RHD research studies that offer patients with transport to facilities
Good patient-provider relations	Any mention of the desire to maintain a good relationship with health workers facilitating continued access to RHD care and adherence to treatment
Improvement on treatment	Any mention of being motivated to seek and stay in RHD care plus following all health recommendation due to the noticeable improvement witnessed in a patient's life
Good care practices by health workers	Any mention of being motivated to seek or to stay on treatment due to the positive treatment received form health workers

Formatted: Position: Horizontal: Center, Relative to: Margin, Vertical: 0", Relative to: Paragraph, Wrap Around

Financial assistance from family	Any mention of being motivated to seek or stay in care due to having people who offer money to facilitate access to RHD care.
Reminders for treatment	Any mention of being reminded for treatment by
Social support	Any mention of the role of social support from relatives, friends, partners, workmates etc. in facilitating treatment seeking and adherence / healthy living
Short distance to facility where care is received	Any mention of being motivated to stay in care due to the short distance traveled to access care

Formatted: Position: Horizontal: Center, Relative to: Margin, Vertical: 0", Relative to: Paragraph, Wrap Around

Appendix 4. COREQ 32-ITEM CHECKLIST

Item #	Guide questions/description	Reported on p. #
**Domain 1: Research team and reflexivity**		
1. Interviewer/facilitator	Which author/s conducted the interview?	4
2. Credentials	What were the researcher's credentials?	4
3. Occupation	What was their occupation at the time of the study?	4
4. Gender	Was the researcher male or female?	4
5. Experience and training	What experience or training did the researcher have?	4
6. Relationship with participants established	Was a relationship established prior to study commencement?	4
7. Participant knowledge of the interviewer	What did the participants know about the researcher?	4
8. Interviewer characteristics	What characteristics were reported about the inter viewer/facilitator?	4
**Domain 2: study design**		
9. Methodological orientation and Theory	What methodological orientation was stated to underpin the study?	4
10. Sampling	How were participants selected?	4
11. Method of approach	How were participants approached?	5
12. Sample size	How many participants were in the study?	5
13. Non-participation	How many people refused to participate or dropped out? Reasons?	5
14. Setting of data collection	Where was the data collected?	3,5
15. Presence of non-participants	Was anyone else present besides the participants and researchers?	5
16. Description of sample	What are the important characteristics of the sample?	5
17. Interview guide	Were questions, prompts, guides provided by the authors?	5
18. Repeat interviews	Were repeat interviews carried out?	5
19. Audio/visual recording	Did the research use audio or visual recording to collect the data?	5
20. Field notes	Were field notes made during and/or after the interview?	5
21. Duration	What was the duration of the interviews?	5
22. Data saturation	Was data saturation discussed?	5

Formatted: Position: Horizontal: Center, Relative to: Margin, Vertical: 0", Relative to: Paragraph, Wrap Around

23. Transcripts returned	Were transcripts returned to participants for comment and/or correction?	5
**Analysis**		
24. Number of data coders	How many data coders coded the data?	5
25. Description of the coding tree	Did authors provide a description of the coding tree?	5
26. Derivation of themes	Were themes identified in advance or derived from the data?	5
27. Software	What software, if applicable, was used to manage the data?	5
28. Participant checking	Did participants provide feedback on the findings?	5
29. Quotations presented	Were participant quotations presented to illustrate the themes/findings? Was each quotation identified?	6-15
30. Data and findings consistent	Was there consistency between the data presented and the findings?	15-20
31. Clarity of major themes	Were major themes clearly presented in the findings?	6-15
32. Clarity of minor themes	Is there a description of diverse cases or discussion of minor themes?	6-15

Formatted: Position: Horizontal: Center, Relative to: Margin, Vertical: 0", Relative to: Paragraph, Wrap Around
